# The Role of Prophylactic HIPEC in High-Risk Gastric Cancer Patients: Where Do We Stand?

**DOI:** 10.3390/cancers17152492

**Published:** 2025-07-28

**Authors:** Alexandros Diamantis, Athina A. Samara, Anastasios Lafioniatis, Michel B. Janho, Theodoros Floros, Konstantinos Tepetes

**Affiliations:** 1 Department of Surgery, University Hospital of Larissa, 41110 Larissa, Greece; alexandrosdoc@hotmail.com (A.D.); tasoslaf95@gmail.com (A.L.); micheljanho@live.co.uk (M.B.J.); tepetesk@gmail.com (K.T.); 2 Department of Radiology, General Hospital of Larissa, 41110 Larissa, Greece; ftheodor93@gmail.com

**Keywords:** HIPEC, high-risk gastric cancer, peritoneal carcinomatosis

## Abstract

For patients diagnosed with a malignancy at high risk of developing peritoneal metastases, the concept of prophylactic hyperthermic intraperitoneal chemotherapy (HIPEC) has emerged. The aim of the present study is to assess the role of prophylactic HIPEC in gastric cancer patients at high risk of PC, based on the currently available data in the literature. In total, 14 RCTs and 16 non-RCTs were identified and included in the present review, with 1383 patients included in the RCTs (627 of whom underwent HIPEC) and 1647 patients included in the non-RCTs (with 609 undergoing HIPEC). Prophylactic HIPEC appears to be useful and effective in treating patients with high-risk gastric cancer, improving both overall and disease-free survival. The heterogeneity of data regarding treatment protocols and complication rates suggests that further research is necessary to develop optimal therapeutic approaches and personalized treatment options; in particular, large-scale randomized control trials are needed in order to elucidate the potential benefits associated with the use of prophylactic HIPEC.

## Simple Summary

The use of hyperthermic intraperitoneal chemotherapy (HIPEC) for microscopic lesions is associated with a significant benefit in patients with peritoneal carcinomatosis (PC). For patients who are diagnosed with a malignancy with high potential to develop peritoneal metastases, the concept of prophylactic HIPEC has emerged. Prophylactic HIPEC appears to be useful and effective in treating patients with high-risk gastric cancer, improving both overall and disease-free survival. The heterogeneity of data regarding treatment protocols and complication rates suggests that further research is needed in order to develop optimal personalized therapeutic approaches. Quality of life also needs to be taken into account, given that it is equally important as the currently applied survival statistics.

## 1. Introduction

The term peritoneal carcinomatosis (PC), which was first used by Sampson in 1931, refers to the cavitary metastatic disease resulting from the infiltration of tumor cells into the peritoneum. It was originally used to describe the involvement of the peritoneum in cases of ovarian cancer [1], although the term was later used to refer to any metastatic peritoneal deposits. PC arises commonly in the context of cancers of the gastrointestinal, gynecological, and genitourinary tracts. Ovarian, colon, and gastric cancers comprise the majority of cases of PC. Other cancers that may result in metastatic involvement of the peritoneum include pancreatic cancer and malignancies of the appendix, small intestine, endometrium, and prostate. Finally, extra-abdominal malignancies such as breast cancer, lung cancer, and malignant melanoma account for approximately 10% of PC cases [2]. Due to the fact that there are no accurate methods for the preoperative diagnosis of PC, epidemiological data regarding the incidence of the disease are inadequate and present wide variations. Female patients comprise the majority of cases, and, in about 70% of cases, the diagnosis is made at the age of 60 years or older [3].

While the pathophysiology of peritoneal metastasis is complex, recent extensive scientific research on the subject has yielded useful insights. PC can be the result of exfoliating tumor cells from intra-abdominal malignancies that circulate in the peritoneal cavity and may appear preoperatively (synchronous PC) or after surgical treatment (metachronous PC). On the other hand, lymphatic and hematogenous spread seem to play an important role in other extra-abdominal cancers. Biological research has shown that there are three types of metastatic spread in the peritoneum: (1) Random local distribution, (2) complete redistribution, and (3) widespread cancer distribution. Knowing the different patterns of metastatic spread can help in determining the optimal surgical approach for each patient [4].

Thanks to the extensive work of Dr. Sugarbaker [5], cytoreductive surgery (CRS)—with the aim of removing macroscopic lesions—in combination with hyperthermic intraperitoneal chemotherapy (HIPEC) for microscopic lesions [6,7] has shown a significant benefit in terms of the quality of life (QoL) of patients with PC. HIPEC refers to the use of a heated solution containing a chemotherapeutic agent inside the peritoneal cavity. The chemotherapeutic agent acts directly on the malignant cells, thus avoiding some of the adverse effects of systemic chemotherapy, in combination with heat to allow for deeper tissue penetration, cytotoxicity, and metabolic alterations of cancer cells [8,9,10,11]. For patients with PC related to appendiceal or colorectal carcinoma, CRS in combination with HIPEC is a first-line treatment [12,13], and it has also shown promise for use in patients with ovarian, gastric, and neuroendocrine tumors [14,15,16].

The aim of the present study is to assess the role of prophylactic HIPEC in gastric cancer patients at high risk of PC, based on the currently available data in the literature.

## 2. High-Risk Gastric Cancer and HIPEC

Patients with advanced stages or unfavorable histological features have a particularly high risk of PC, leading to a significant reduction in treatment results and high postoperative recurrence rates. In this context, for patients who are diagnosed with a malignancy with high potential to develop peritoneal metastases, the concept of prophylactic HIPEC has emerged. A recent study using prophylactic HIPEC in patients with high-risk gastric cancer showed not only an improvement in perioperative results but also survival rates, suggesting that the integration of prophylactic HIPEC could be beneficial in reducing peritoneal recurrence when compared to conventional adjuvant treatments [17]. However, it is important to underline the great importance of carefully selecting patients who may benefit from the procedure and that multidisciplinary collaboration is needed in order to develop protocols for their personalized evaluation and treatment. At the same time, the use of real-time thermal imaging may help to optimize the infusion technique, thereby delivering maximum efficiency while also avoiding damage to the surrounding healthy tissues. Moreover, combinations with other approaches, such as immunotherapy and targeted agents, should be considered as possible treatment options in the context of an individualized approach and need to be studied further in the future.

In a comprehensive systematic review evaluating the effects of prophylactic HIPEC in terms of overall survival and disease-free survival in patients with high-risk gastric cancer, it was concluded that overall survival was significantly higher and recurrence rates were lower in patients receiving prophylactic HIPEC [18]. Similarly, a descriptive review has reported that patients receiving prophylactic HIPEC in addition to surgery exhibited lower morbidity than those that were treated only with surgery; at the same time, overall survival at 3 and 5 years was greater for patients that received HIPEC. These results highlight the importance of HIPEC in terms of disease-free survival when compared to surgery alone.

In accordance with the above, a multicentric, randomized, controlled open-label phase III study that included 200 patients showed improved 2- and 5-year survival rates in the HIPEC arm when compared with patients that were treated with surgery alone and adjuvant chemotherapy using the FLOT9 protocol [19]. Moreover, peritoneal recurrence was significantly lower in the HIPEC cohort, suggesting that prophylactic HIPEC may exert a beneficial impact by preventing peritoneal dissemination of tumor cells. It should be remembered that, although prophylactic HIPEC is sometimes associated with an increased risk of postoperative complications, the long-term benefits—including reduced recurrence rates and enhanced survival metrics—may outweigh these risks. Several studies have reported manageable adverse effects, which have led to refinements in treatment protocols designed to optimize patient safety and recovery. Advances in surgical oncology are continually evolving to improve the application of HIPEC. Techniques such as incomplete CRS combined with HIPEC have been investigated, suggesting potential benefits in selected patients even when complete resection is not achievable. In terms of progress, research aims to refine patient selection criteria, biomarkers for response, and HIPEC administration modalities—such as the timing and selection of medicines—in order to maximize its therapeutic efficacy while minimizing toxicity.

Moreover, a retrospective case–control study that included 44 patients concluded that prophylactic HIPEC followed by adjuvant therapy resulted in an increase in disease-free survival by deterring peritoneal recurrence in carefully selected gastric cancer patients [20]. The clinical implementation of HIPEC requires meticulous planning, which involves coordinated efforts between surgical oncologists, medical oncologists, and specialized nursing staff. Emerging protocols advocate for the use of enhanced recovery after surgery (ERAS) protocols together with EPEC, allowing for a reduction in postoperative morbidity and accelerated recovery times. These protocols are essential to ensure that high-risk patients receive proper support during treatment, highlighting the need for integrated care models that integrate nutritional support, psychological advice, and standardized postoperative monitoring. Similarly, a randomized cohort study that included 80 patients with locally advanced gastric cancer reported better short-term postoperative outcomes, significantly better disease-free survival, and lower peritoneal recurrence in the HIPEC plus D2 Gastrectomy cohort when compared to the surgery-only cohort [21].

The dragon II trial is a multicentric Phase III randomized controlled study that investigated the safety and efficacy of the combination of neoadjuvant laparoscopic HIPEC (NLHIPEC) with NAC in the neoadjuvant phase, followed by curative surgery with intraoperative HIPEC, in turn followed by adjuvant chemotherapy (AC) [22]. This study emphasizes the need to stratify patients based on their unique clinical and genetic profiles, which facilitates more precise cancer treatments. By evaluating factors such as tumor biology, patient comorbidities, and previous treatment responses, oncologists can develop HIPEC protocols with the intent of maximizing therapeutic results while minimizing possible adverse effects. The evidence provided in this study, together with previous research, suggests that a personalized multidisciplinary approach may not only improve the efficacy of HIPEC but could also contribute to a better QoL for patients undergoing treatment. Recent studies have indicated that prophylactic HIPEC can lead to improved survival rates and reduced recurrence in selected patient populations [23,24]. This is particularly salient for patients diagnosed with locally advanced gastric cancer who present a significant risk of peritoneal metastasis. Evidence suggests that HIPEC, when administered intra-operatively after tumor resection, may significantly decrease microscopic residual disease—one of the main contributors to postoperative recurrence.

A meta-analysis included 22 studies (12 of which were RCTs) with a total of 2097 patients. It was found that the 1-, 3-, and 5-year overall survival rates were significantly more favorable in the HIPEC patient group (OR 5.10, 2.07, and 1.96, respectively). Compared with the control group, the overall and peritoneal recurrence rates in the HIPEC group were significantly lower. HIPEC was also significantly favorable with respect to the control group in terms of renal dysfunction- and pulmonary dysfunction-related complications (OR 0.41 and 0.24, respectively) [25]. Another meta-analysis including 12 studies pointed out that CRS combined with HIPEC may lead to better prognosis for patients suffering from locally advanced gastric cancer in both prophylactic and curative settings. However, due to significant postoperative morbidity and mortality rates, careful patient selection is extremely important to achieve the best results [26].

## 3. Prophylactic HIPEC in High-Risk Gastric Cancer

A comprehensive search of the available literature regarding the role of prophylactic HIPEC in high-risk gastric cancer patients was conducted using the online databases Medline (Pubmed) and Scopus. The MeSH terms used were “HIPEC,” “prophylactic” and “gastric cancer.” A manual search of related articles was also carried out.

**Figure 1:**
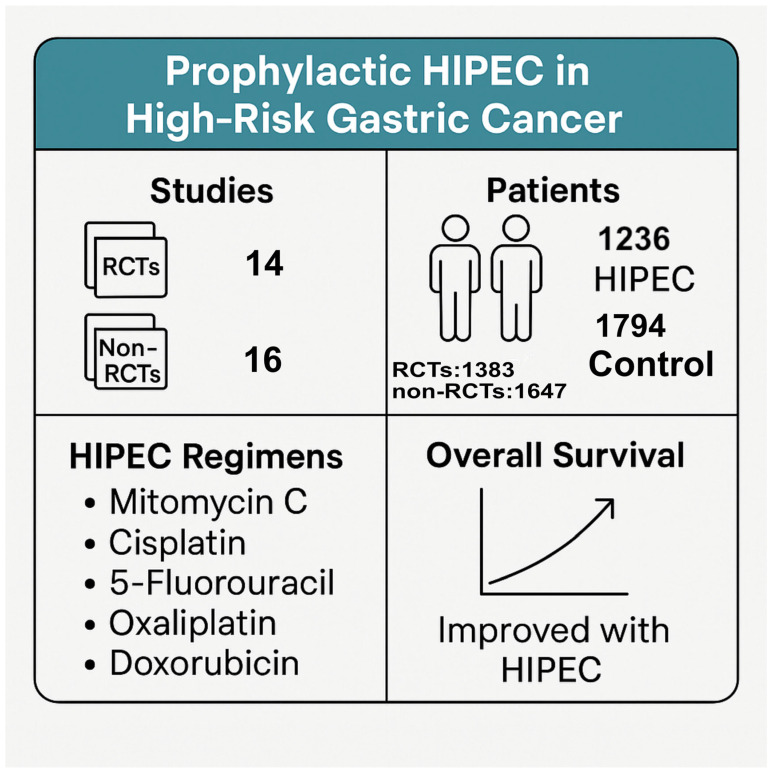
Schematic summary of the review results.

Regarding the use of HIPEC as a prophylactic tool in selected high-risk gastric cancer patients, 14 RCTs [21,27,28,29,30,31,32,33,34,35,36,37,38,39] and 16 non-RCTs [20,40,41,42,43,44,45,46,47,48,49,50,51,52,53] were identified. The main characteristics of the included RCTs are displayed in Supplementary Table S1, and those of the non-RCTs are displayed in Supplementary Table S2. In total, of the 1383 patients included in the RCTs, 627 of them underwent HIPEC (with the rest serving as controls), and, of the 1647 patients included in the non-RCTs, 609 of them underwent HIPEC (Figure 1). Table 1 summarizes the main outcomes of the RCT studies, while Table 2 presents the respective results from the non-RCT studies.

In the 14 RCTs with a minimum total number of 50 patients, the use of prophylactic HIPEC was investigated in combination with radical surgery (RS) or cytoreductive surgery (CRS) in patients with high-risk gastric cancer. These studies used a range of chemotherapeutic agents—including mitomycin C (MMC), cisplatin (CIS), 5-fluorouracil (5-FU), oxaliplatin, and doxorubicin—delivered at intraperitoneal temperatures between 40 °C and 45 °C for durations varying from 30 to 120 min. In terms of overall survival (OS), most studies demonstrated a significant benefit in the HIPEC group compared to the controls. Koga et al. [27] and Kaibara et al. [28] reported the most promising results, with higher 3- and 5-year OS rates in the HIPEC group (83.0% vs. 67.3% and 71.5% vs. 59.7%, respectively). Similar benefits were noted by Hamazoe et al. [29] (5-year OS: 64% vs. 52%) and Yonemura et al. [32] (5-year OS: 61% vs. 42%). Notably, Deng et al. [34] reported a 3-year OS of 59.09% in the HIPEC group versus only 34.15% in controls, and Cui et al. [36] found a significant difference of 75% versus 35.41% at 3 years. Kuramoto et al. [33] observed a 5-year survival rate of 43.8% in the HIPEC group, with no survivors in the control group. Similarly, Rudloff et al. [37] reported a median survival of 11.3 months with HIPEC versus no survivors at 12 months in the control arm, and Beeharry et al. [21] also demonstrated a strong advantage in overall survival (3-year OS: 93% vs. 65%). On the other hand, Fan et al. [39] reported high rates of survival in both arms, suggesting that prophylactic HIPEC with cisplatin was safe and tolerable but did not reduce the risk of peritoneal recurrence.

Moreover, 16 non-RCTs with a minimum of 40 patients investigated the same subject, which were included in the present analysis. In a similar manner, they employed a wide range of chemotherapeutic regimens—including MMC, CIS, etoposide, doxorubicin, oxaliplatin, paclitaxel, and lobaplatin—delivered at intraperitoneal temperatures between 40 °C and 44 °C for durations ranging from 30 to 90 min. Several studies demonstrated a clear benefit in OS with the addition of HIPEC. Kang et al. [43] reported a significant improvement in 5-year OS (43% in the HIPEC group versus just 10%). Additionally, Rosa et al. [53] found a 5-year OS of 33% in the HIPEC arm compared to 9% in controls, and Zhong et al. [51] reported favorable 3-year OS outcomes using lobaplatin-based HIPEC (89.4% versus 84.3%). Rau et al. [48] showed a 3-year OS of 17.5% in the HIPEC group, while no patients survived in the control group, and Bonnot et al. [47] also demonstrated a generally poor 5-year OS; however, the HIPEC group showed a certain improvement (10.82% compared to 6.43%). Other studies have highlighted improved median or mean survival, with Hultman et al. [42] reporting a mean survival of 17.4 months versus 11.1 months in controls, Boerner et al. [46] finding a median survival of 17.2 months versus 11.0 months, and Coccolini et al. [20] observing an increase in mean survival from 27.1–28.2 months in controls to 36.6 months with HIPEC. In contrast, some studies showed less favorable or inconsistent outcomes. Kunisaki et al. [40] reported a slightly lower 5-year OS in the HIPEC group (49%) compared to controls (56%). Similarly, Diniz et al. [52] observed higher 5-year OS in the control group (68.7% versus 59.5%), although disease-free survival (DFS) was better in the HIPEC group (65.8% vs. 49.5%). Xie et al. [49] and Zhu et al. [50] presented relatively balanced outcomes between HIPEC and control groups in terms of both DFS and OS, suggesting that patient selection, disease burden, and treatment context may heavily influence the observed outcomes.

## 4. Challenges and Considerations

**Figure 2:**
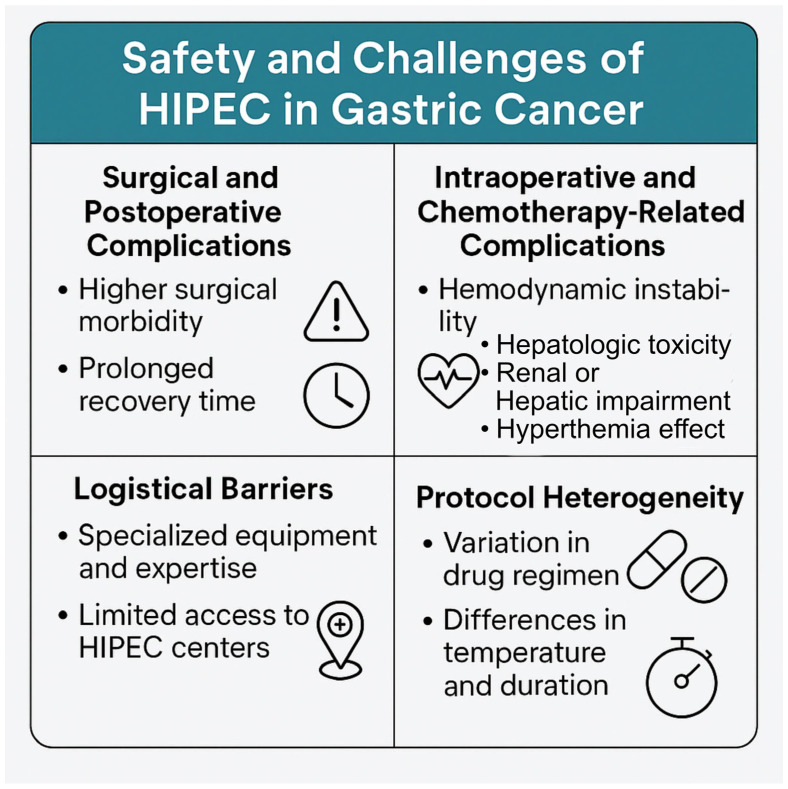
Challenges and considerations regarding the use of HIPEC.

The application of HIPEC in gastric carcinoma patients, while promising in terms of its potential to improve patient outcomes, is accompanied by a series of safety problems and challenges that require careful consideration (Figure 2). The recent literature has highlighted various complications that can arise during and after the administration of HIPEC, underlining the need for a more complete understanding of its feasibility in clinical environments [54,55,56,57]. In a retrospective analysis, it was shown that the incidence of surgical complications in patients undergoing HIPEC was significantly higher than that in those receiving traditional surgical approaches alone. The authors pointed out that the additional intraoperative time required for HIPEC can exacerbate these risks, leading to longer periods of recovery accompanied by greater morbidity [56]. In fact, this is in concordance with an earlier report noting that postoperative complications directly influence the duration of hospitalization and the overall QoL for patients who recover from gastric resections with HIPEC [57]. Moreover, the postoperative recovery process imposes its own series of challenges. Studies have indicated that patients undergoing prophylactic HIPEC may experience prolonged recovery times, which can affect their ability to resume normal activities and decrease the overall satisfaction of the patient with the surgical outcome. These prolonged recovery periods can be attributed to both the surgical technique and the effects of the chemotherapeutic agents used, particularly when patients have an advanced disease or a poor state of performance before the intervention. The multi-factorial nature of these recovery results requires further research to establish a measure of postoperative assistance that is tailored to patients with gastric carcinoma who have received HIPEC. There is an exaggerated metabolic and inflammatory response after surgery, which may be considered to be physiological in view of the nature of surgery combined with the use of HIPEC [58].

Intraoperative complications during CRS plus HIPEC are multifaceted, involving factors such as hemodynamic instability, intraoperative metabolic changes, significant blood loss, organ injury, hyperthermia-related complications, wound dehiscence, increased risk of infection risk and technical challenges [59]. Moreover, major anesthetic problems can arise, including hemodynamic instability, respiratory complications, renal failure, coagulation abnormalities, and thermal management issues. Effective perioperative management necessitates enhanced monitoring, meticulous hydration and temperature management, and a multidisciplinary approach to address problems and improve patient outcomes [59]. Chemotherapy-related and systemic complications have also been identified after HIPEC. Hematologic toxicity—such as neutropenia and thrombocytopenia—is a well-recognized complication, especially in elderly patients [60]. The chemotherapeutic agents used in HIPEC (e.g., high-dose cisplatin) can cause nephrotoxicity, leading to renal and liver impairment [59].

It needs to be mentioned that the operational feasibility of integrating HIPEC into standard treatment protocols for patients with high-risk gastric carcinoma remains a subject of debate. The logistical challenges associated with the technique, including the need for specialized equipment and expert staff, can limit its availability in routine clinical settings. As these challenges vary significantly between institutions, access to prophylactic HIPEC can become uneven, thus influencing the selection and results of patients between different populations. Consequently, dealing with these logistical concerns is essential to improve the adoption of HIPEC as a standard treatment mode [57].

A further layer of complexity arises due to the heterogeneity in treatment protocols. The variations in the administration of HIPEC—such as differences in the chemotherapeutic agents used, duration and temperature of the perfusion, and patient selection criteria—can further complicate the interpretation of the associated results [26]. This inconsistency underlines the need for multicenter clinical studies aimed at establishing evidence-based guidelines, including optimal protocols for the prophylactic application of HIPEC for the management of gastric cancer. In turn, this progress could potentially help to resolve safety problems and improve patient recovery trajectories.

Hyperthermia is selectively lethal for malignant cells and can act synergistically with other anticancer treatments, including chemotherapy and radiotherapy [60,61]. There is considerable heterogeneity in the extent, timing, and underlying mechanisms regarding the thermal enhancement of chemotherapy. Synergism with heat is particularly evident for platinum compounds and mitomycin C, while other agents—such as taxanes and antimetabolites—do not present thermal enhancement effects. Third, hyperthermia improves tissue perfusion and oxygenation, and it may enhance tissue penetration [61]. Other drugs showing increased tumor penetration when combined with hyperthermia include carboplatin, oxaliplatin, and doxorubicin [62]. Interestingly, hyperthermia may diminish the systemic toxicity of some drugs, such as doxorubicin and cyclophosphamide, by increasing their alkylation and/or excretion [63]. Besides the choice of drug, the length of exposure to hyperthermia might also play a crucial role. The ideal target temperature for HIPEC is unknown. Although it is known that DNA repair is inhibited at temperatures >41 °C in vitro, the relationship between temperature and anticancer agent efficacy in vivo is not known. Additionally, due to the heat sink effect of the tumor blood vessels, the actual tissue temperature that can be reached is lower than that of the heated IP solution. Hyperthermia elicits the expression of heat shock proteins (HSPs), which have been shown to exert anti-apoptotic and proliferative effects, as well as inducing resistance to chemotherapy [64,65]. Furthermore, temperatures above 41 °C may cause scald injury to the peritoneum, which is already extensively damaged by the CRS [60].

Anastomotic leak and perforation of the gastrointestinal tract are the most dangerous complications following CRS–HIPEC, which have been linked with significant postoperative morbidity and mortality. Nogueiro et al. [65] identified advanced age, increased PCI, cisplatin dose >240 mg during HIPEC, and the presence of colorectal anastomosis as independent risk factors for anastomotic leak. Meanwhile, for perforation, male gender and intraoperative red blood cell transfusions were the only independent risk factors identified [65].

In summary, while prophylactic HIPEC can be considered as a new approach to mitigate the risk of peritoneal carcinomatosis in patients with high-risk gastric carcinoma, it is essential to navigate safety problems and the accompanying challenges. The interactions between surgical complications, the dynamics of recovery, and logistical feasibility must be examined in depth in order to ensure that the potential benefits of HIPEC are translated into significant improvements in the care of patients and clinical results.

## 5. Quality of Life Following HIPEC

Quality of life (QoL) is a multidimensional, dynamic and subjective value that focuses on the patient’s perspective, including their physical, functional, emotional and social well-being. In this context, assessing QoL is an outcome of great importance when evaluating the full impact of a disease on patients and their families [66]. CRS and HIPEC can impact postoperative QoL in multiple ways, affecting physical, emotional, social, and functional domains. In the immediate postoperative period, patients commonly experience declines in global and physical QoL as a result of pain, gastrointestinal dysfunction, fatigue, and emotional distress [67]. Most studies report that global and physical QoL immediately deteriorate significantly in the postoperative period, with a gradual return to 80% to 100% of baseline between 6 and 12 months—although some patients may recover as early as 3 months or as late as 24 months [68,69,70]. Emotional well-being often recovers more quickly than physical or social functioning, with some studies observing improvements in emotional QOL as early as three months post-operatively. Persistent depressive symptoms, however, remain prevalent in up to one-third of patients at even a year or more after surgery. Social QOL tends to decline more subtly and may take longer to recover, with some large studies indicating incomplete recovery even at 24 months. Functional status—including return to work and daily activities—often mirrors physical recovery, but long-term disability or limited capacity persists in a subset of patients, especially those with more aggressive disease [71].

Validated instruments such as the SF-36, FACT-G, and EORTC QLQ-C30 are commonly used to assess QOL, with additional tools like the Brief Pain Inventory (BPI), CES-D and ECOG scale used to capture specific domains [71]. Long-term survivors demonstrate varied outcomes: while many regain their baseline or acceptable QoL levels, a considerable number report ongoing fatigue, sleep disturbances, and gastrointestinal symptoms even years after surgery [72]. Pain generally improves or returns to baseline by 12 months postoperatively, especially in those with complete cytoreduction [73]. Nevertheless, persistent somatic symptoms—particularly fatigue, insomnia, and bowel dysfunction—are frequently reported; furthermore, sexual dysfunction may be under-recognized in this population. Cross-sectional and prospective studies alike suggest that although CRS/HIPEC is associated with an initial decline in QoL, most patients experience gradual and meaningful recovery, underscoring the importance of individualized supportive care and long-term follow-up to address lingering physical and psychosocial challenges [71].

## 6. Future Perspectives

Recent progress in surgical oncology has considerably influenced the application of HIPEC in high-risk gastric cancer patients. Innovations in surgical techniques and technology have improved the administration and overall efficiency of the procedure, with the ultimate aim of improving patient outcomes. One aspect of this progress is the adoption of mini-invasive approaches, such as HIPEC assisted by laparoscopy. Studies have indicated that laparoscopy not only improves recovery times but also reduces postoperative complications when compared to traditional open surgical methods [74]. This change to mini-invasive techniques is particularly relevant for patients with gastric cancer, who often have complex medical histories that may complicate conventional surgical interventions.

Recent technological improvements, including the development of infusion systems that allow for a more uniform distribution of chemotherapeutic agents, have been essential in optimizing HIPEC delivery [75]. These improvements facilitate controlled and targeted application of the heated drugs, which can lead to improved pharmacodynamic responses and reduced systemic toxicity. In addition, the integration of real-time imaging techniques during HIPEC has shown promise in allowing surgeons to monitor distribution models and adjust infusion dynamics accordingly, thus refining the efficiency of treatments.

Another notable advancement lies in the combination of neoadjuvant chemotherapy with HIPEC. This therapeutic strategy aims to reduce the tumor load before surgery and maximize the effectiveness of perioperative treatments. The recent literature has stressed that the administration of neoadjuvant chemotherapy in conjunction with HIPEC can lead to significant improvements in surgical results. Specifically, studies have reported higher rates of R0 when combining these methods, supporting their synergistic potential [63]. In addition, assessment of the effects on pathological response rates has opened new paths toward understanding the biological interactions between chemotherapeutic agents and the tumor microenvironment in gastric cancer patients.

While surgical oncology continues to evolve, guidelines based on evidence advocating for the incorporation of these technologies and methodologies are emerging. This includes not only the technical aspects regarding the realization of HIPEC but also the stratification of patients on the basis of risk factors, which can shed light on their eligibility for neoadjuvant therapy followed by HIPEC. The creation of multidisciplinary teams, including surgical oncologists, medical oncologists, and radiologists, is increasingly recognized as essential for the development of individualized treatment plans that exploit the latest surgical and pharmacological advances [75].

Research on the molecular and genetic foundations of gastric cancer can guide the development of targeted therapies that complement HIPEC protocols. Personalized medicine approaches, involving the adaptation of treatment modalities based on specific tumor characteristics in individual patients, are expected to further refine the effectiveness of HIPEC. The incorporation of biomarkers and genetic profiling can help in predicting responses to treatment and optimizing the selected regimen, thus potentially improving overall survival rates and the quality of life in patients with high-risk gastric cancer.

Treatment protocols involving prophylactic HIPEC require careful patient selection, usually incorporating advanced imaging techniques and biomarkers to stratify patients according to their risk profiles [24]. Such precision medicine approaches are essential to maximize the therapeutic efficacy of HIPEC, while mitigating possible adverse effects associated with this intensive treatment modality. Notably, variations in HIPEC administration—such as the choice of medicines, doses, and maintenance of temperature—have been the focus of recent investigations, highlighting the need for standard protocols to ensure consistency in results under different clinical configurations.

The implications of prophylactic HIPEC extend beyond mere survival metrics; it also covers the broader spectrum of the patient’s quality of life. As has been reported recently [23], specific patient factors such as psychosocial well-being and functional status remain critical considerations in the evaluation of a treatment’s effectiveness. Consequently, future research should not only emphasize survival statistics but should also integrate qualitative assessments that address results reported by the patient, such as their emotional health and general satisfaction after treatment. Innovations such as robotic-assisted surgery, augmented reality in surgical navigation, and real-time molecular imaging during HIPEC delivery can increase the accuracy of the procedure and further reduce complications. In addition, the integration of personalized medicine approaches—particularly through genomic profiling and directed therapies—has transformative potential to increase the benefits of prophylactic HIPEC in high-risk populations.

The 2024 Peritoneal Surface Oncology Group International (PSOGI) Delphi consensus [76] provides comprehensive recommendations for the standardized use of HIPEC, particularly in gastric cancer patients with peritoneal surface malignancy. These guidelines aim to enhance consistency in clinical practice and improve comparability across future studies. For patient selection, HIPEC is advised only when complete or near-complete cytoreduction is achievable, thus ensuring that the maximal benefit is obtained. Chemotherapeutic regimens should be based on body surface area, with oxaliplatin (200 mg/m^2^) and cisplatin considered among the preferred agents, often combined with 5-FU or other adjuncts. Perfusion techniques should maintain an intraperitoneal temperature between 41 and 42 °C, monitored using at least three temperature probes, with a perfusion duration of 90 min having been identified as optimal. Intraoperative parameters also include standardized drug dosing, consistent use of carrier solutions, and careful thermal control to ensure homogeneous distribution.

Before the clinical appraisal of our results, several limitations of the present review should be considered. First of all, as a review article, the quality of our research is based on the quality of the original included studies. A major issue is the fact that there is great heterogeneity regarding the HIPEC protocols that were used in each study, differences in patient selection, and a lack of standardized techniques. Moreover, the lack of large-scale RCTs regarding the outcomes of prophylactic HIPEC, particularly in terms of survival and postoperative quality of life, remains a barrier to drawing sound conclusions.

## 7. Conclusions

Prophylactic HIPEC appears to be useful and effective in treating patients with high-risk gastric cancer, improving their overall and disease-free survival. The heterogeneity of data regarding treatment protocols and complication rates suggests that further research is needed in order to develop optimal therapeutic approaches that better suit individual patients. In the rapidly evolving field of oncology, this relates to the integration of a variety of emerging biomarkers, the in-depth understanding of the biological behavior of high-risk gastric cancers, the evaluation of different surgical and imaging modalities, the multidisciplinary collaboration of different medical specialists, and the development of large multicenter randomized clinical trials. Quality of life should also be taken into account, given that it is equally important as the currently applied survival statistics. Future research should aim to define optimal approaches for the stratification of patients and the selection of therapeutic strategies, such that the treatment effect is maximized in an individualized manner while avoiding potential side effects and complications. Rigorous research is therefore needed in order to support each patient in the best way possible.

## Figures and Tables

**Table 1:** Outcomes of RCTs regarding prophylactic HIPEC.

	Intervention							DFS		OS	
Author	HIPEC	Control Group	NAC	AC	HIPEC Duration (min)	Drug	Temp (°C)	HIPEC	Control	HIPEC	Control
Koga et al. [27]	HIPEC + RS	RS	ND	ND	50–60	MMC	44–45			3-year: 83.0%	67.30%
Kaibara et al. [28]	HIPEC + RS	RS	ND	ND	50–60	MMC	44–45			5-year: 71.5%	59.70%
Hamazoe et al. [29]	HIPEC + RS	RS	ND	ND	50–60	MMC	40–45			5-year: 64%	52%
Ikeguchi et al. [30]	HIPEC + RS	Systemic chemotherapy	No	Yes		MMC	44–45	30 months	23 months		
Fujimoto et al. [31]	HIPEC + RS	RS	ND	Yes	120	MMC	43–44			4-year: 76%	58%
Yonemura et al. [32]	HIPEC + RS	RS	ND	Yes	60	MMC/CIS	42–43			5-year: 61%	42%
Kuramoto et al. [33]	HIPEC + RS	RS	ND	Yes	60	CIS				5-year: 43.8%	0%
Deng et al. [34]	HIPEC + RS	RS	ND	Yes	60–90	MMC/5-FU	42–43			3-year: 59.09%	34.15%
Zhang et al. [35]	HIPEC + CRS	CRS	NR	NR	60–90	MMC/CIS	42.5–43.5			32-month survival: 14.7%	2.9%
Cui et al. [36]	HIPEC + RS	RS	YES	YES	90	CIS/5-FU	41–43			3-year: 75%	35.41%
Rudloff et al. [37]	HIPEC + CRS	Systemic chemotherapy	NR	NR	30	Oxaliplatin	41			11.3 months median survival	All dead within 12 months
Reutovich et al. [38]	HIPEC + RS	RS	ND	ND	60	CIS/doxorubicin	42	3-year progression-free survival: 47% (95% CI 36–61)	3-year progression-free survival: 27% (95% CI 17–43)		*p* = 0.0024.
Beeharry et al. [21]	HIPEC + RS	RS	ND	Yes	60	CIS	42	3-year: 93%	65%		
Fan et al. [39]	HIPEC + RS	RS	ND	Yes	30	CIS	42.5–43			3-year: 87.9%	100%

**Table 2:** Outcomes of non-RCTs regarding prophylactic HIPEC.

	Intervention							DFS		OS	
Author	HIPEC	Control Group	NAC	AC	HIPEC Duration (min)	Drug	Temp (°C)	HIPEC	Control	HIPEC	Control
Kunisaki [40]	HIPEC + RS	RS		yes	40	MMC/CIS/etoposide	42–43			5-year: 49%	56%
Li [41]	HIPEC + CRS	CRS			60	MMC/CIS	40–45				
Hultman [42]	HIPEC + CRS	Systemic chemotherapy	Yes		90	CIS/doxorubicin	42–44			17.4 months mean survival	11.1 months
Kang [43]	HIPEC + RS	RS	ND	Yes	60	MMC/CIS/etoposide	41–4	3-year: 66.03%	28.87%	5-year: 43%	10%
Yarema [44]	HIPEC + RS	RS	ND	ND	90	MMC	41–43.5			1-year: 100%/	12 months/52.6% 1 year
	HIPEC + CRS	Systemic chemotherapy		Yes	90	MMC	41–43.5			1-year: 68.8%	25%
Kim [45]	HIPEC + CRS	CRS			90	MMC	41				
Coccolini [20]	HIPEC + RS	RS	Yes	ND	90	CIS/paclitaxel	40–41	34.5 months	21.6–27.7	36.6 months mean survival	27.1–28.2
Boerner [46]	HIPEC + CRS	Systemic chemotherapy	NR	NR	60	CIS/doxorubicin	42–43			17.2 median survival	11.0 median survival
Bonnot [47]	HIPEC + CRS	CRS	Yes	Yes	30–90	MMC/CIS/oxaliplatin	42–44			5-year: 10.82%	5-year: 6.43%
Rau [48]	HIPEC + CRS	Systemic chemotherapy	Yes	Yes	60	MMC/CIS	41			3-year: 17.5%	0%
Xie [49]	HIPEC + RS	RS	ND	Yes	60	CIS	42–43	3-year: 63%	60%	3-year: 68%	66%
Zhu [50]	HIPEC + RS	RS	ND	Yes	60	CIS	41.5–42.5	36.5 months	24.5	NR	33 months
Zhong [51]	HIPEC + RS	RS	ND	Yes	60	Lobaplatin	43	3-year: 89.4%	73.90%	3-year: 89.4%	84.30%
Diniz [52]	HIPEC + RS	RS	Yes	Yes	90	MMC	41–42	5-year: 49.5%	65.80%	5-year: 59.5%	68.70%
Rosa [53]	HIPEC +CRS	RS	ND	ND	90	MMC	41–42	5-year: 30%	9%	5-year: 33%	9%

## Supplementary Materials

The following supporting information can be downloaded at: https://www.mdpi.com/article/10.3390/cancers17152492/s1, Table S1. Basic characteristics of RCTs regarding prophylactic HIPEC in high-risk gastric cancer patients. Table S2. Basic characteristics of non-RCTs regarding prophylactic HIPEC in high-risk gastric cancer patients.
