# Supplementary material for: The Role of Prophylactic HIPEC in High-Risk Gastric Cancer Patients: Where Do We Stand?
Source: Cancers (Basel). 2025 Jul 28;17(15):2492. doi: 10.3390/cancers17152492 (PMC12346235; doi:10.3390/cancers17152492)
Supplement: Supplementary file 1 [file cancers-17-02492-s001.zip › cancers-3720868-supplementary.pdf]

Supplementary Materials

Table S1. Basic characteristics of RCTs regarding prophylactic HIPEC in high-risk gastric cancer patients.

						**n**		**Age [mean]**		**Sex [m/f]**		
**Author**	**Country**	**Year**	**Study Period**	**Study Type**	**Total N**	HIPEC	Control	HIPEC	Control	HIPEC	Control	Inclusion Criteria
**Koga** [27]	Japan	1988	1983-1985	RCT	60	32	28					cT3-cT4
**Kaibara [**28]	Japan	1989	1983-1985	RCT	82	42	40					cT3-cT4
**Hamazoe** [29]	Japan	1994	1983-1986	RCT	82	42	40					cT3-cT4
**Ikeguchi** [30]	Japan	1995	1980-1989	RCT	174	78	96			43/35	64/32	cT3
**Fujimoto** [31]	Japan	1999	1987-1996	RCT	141	71	70					cT3-cT4
**Yonemura** [32]	Japan	2001		RCT	139	48	47					cT2-cT4
**Kuramoto** [33]	Japan	2009	1995-2005	RCT	59	30	29	63.4	65.2	13/17	13/16	cT4
**Deng** [34]	China	2009	2002-2004	RCT	85	44	41	NR	NR	NR	NR	cT4
**Yang** [35]	China	2011	2007-2010	RCT	68	34	34	50	51	16/18	19/15	GCPC
**Cui** [36]	China	2014	2006-2010	RCT	192	48	48	53	56	22/26	21/27	cT4
**Rudloff** [37]	USA	2014	2009-2012	RCT	17	9	7	45	52	6/3	4/4	GCPC
**Reutovich** [38]	Belarus	2019	2008-2016	RCT	154	76	78	56	56	50/26	45/33	cT4
**Beeharry** [39]	China	2019	2014-2015	RCT	80	40	40	59	58	23/17	23/17	cT3-cT4
**Fan** [40]	China	2021	2015-2016	RCT	50	33	17	61	60	27/6	14/3	cT3-cT4

Table S2. Basic characteristics of non-RCTs regarding prophylactic HIPEC in high-risk gastric cancer patients.

						**n**		**Age [mean]**		**Sex [m/f]**			
Author	Country	Year	Study Period	Study Type	Total N	HIPEC	Control	HIPEC	Control	HIPEC	Control	Inclusion Criteria	Disease
Kunisaki [41]	Japan	2002	1992-1999	NRCT	124	45	79	53	64.4	32/13	58/21	cT3-cT4	Locally advanced GC
Li [42]	China	2010	1992-2002	NRCT	128	10	44	49.4	55.4	5/5	15/29	GCPC	GC with PC
Hultman [43]	Sweden	2012	2005-2007	NRCT	20	10	10	59	62	5/5	7/3	GCPC	GC with PC
Kang [44]	China	2013	2002-2010	NRCT	112	29	83					cT4	AGC without PC
Yarema [45]	Ukraine	2014	2008-2012	NRCT	49	19	19	NR	NR	15/4	13/6	cT4	AGC without PC
					49	20	20	NR	NR	10/10	16/4	GCPC	GC with PC
Kim [46]	USA	2014	2000-2011	NRCT	112	9	17					GCPC	GC with PC
Coccolini [47]	Italy	2016	2010-2015	NRCT	44	6	28	60.15	72.5	2/4	18/10	pT3–pT4	GC with PC
Boerner [48]	Germany	2016	2006-2013	NRCT	65	38	27					GCPC	GC with PC
Bonnot [49]	France	2019	1989-2014	NRCT	277	180	97	51.13	59.82	83/97	49/48	GCPC	GC with PC
Rau [50]	Germany	2019	2008-2017	NRCT	77	58	19	53.9	57.8	26/32	7/12	GCPC	GC with PC
Xie [51]	China	2020	2014-2017	NRCT	113	51	62	60.9	61.5	36/15	43/19	cT4	AGC without PC
Zhu [52]	China	2020	2018-2019	NRCT	43	22	21					cT3-cT4	AGC without PC
Zhong [53]	China	2020	2016-2017	NRCT	129	61	68	52.4	53.1	32/29	33/35	cT3-cT4	AGC without PC
Diniz [54]	Brazil	2020	2006-2017	NRCT	269	28	241					cT3-cT4	AGC without PC
Rosa [55]	Italy	2021	2006-2015	NRCT	85	23	39	58	68	11/12	20/19	cT2-cT4	AGC without PC
